# The prevalence of gestational diabetes mellitus before and after the implementation of the universal two-child policy in China

**DOI:** 10.3389/fendo.2022.960877

**Published:** 2022-08-18

**Authors:** Hui Zhu, Zhijia Zhao, Jin Xu, Yanming Chen, Qiong Zhu, Liming Zhou, Jie Cai, Lindan Ji

**Affiliations:** ^1^ Department of Internal Medicine, School of Medicine, Ningbo University, Ningbo, China; ^2^ Department of Preventive Medicine, School of Medicine, Ningbo University, Ningbo, China; ^3^ Zhejiang Key Laboratory of Pathophysiology, School of Medicine, Ningbo University, Ningbo, China; ^4^ Department of Pediatrics, Affiliated People’s Hospital of Ningbo University, Ningbo, China; ^5^ Center for Reproductive Medicine, Ningbo Women and Children’s Hospital, Ningbo, China; ^6^ Department of Biochemistry, School of Medicine, Ningbo University, Ningbo, China

**Keywords:** advanced maternal age, gestational diabetes mellitus, universal two-child policy, risk factor, interrupted time series

## Abstract

**Background:**

After the universal two-child policy has been fully implemented, challenges regarding pregnancy complications seemed to be more severe in China. This study aimed to evaluate the prevalence of gestational diabetes mellitus (GDM) and the main risk factors for GDM before and after the implementation of the universal two-child policy in China.

**Methods:**

A retrospective study was performed with 128,270 pregnant women who delivered at Ningbo Women & Children’s Hospital from January 2010 to December 2020. Univariate and multivariate logistic regression analysis was applied to estimate the risk factors associated with GDM prevalence. Segmented regression analyses of interrupted time series (ITS) were conducted to assess the effect of the universal two-child policy on the trends of GDM.

**Results:**

The prevalence of GDM increased remarkably from 4% in 2010 to 21% in 2020. ITS analysis presented that the prevalence of GDM increased by 0.190% (β1) per month from 2010 to 2016 (*P*<0.05), and by 0.044% (β1+β3) per month after the implementation of the universal two-child policy; the rate of elevation of GDM slowed down significantly (β3=-0.146, *P*=0.004). Advanced maternal age (>30 years), multigravidity, multiparity, multiple gestation and gestational hypertension were significantly associated with GDM. Advanced age remained an independent risk factor for GDM even after cross stratification with gravidity and parity. The proportion of women with advanced maternal age (>30 years) increased by 0.161% per month before the implementation of the universal two-child policy and increased by 5.25% during the policy took effect month, and gradually increased by 0.124% (β1+β3) per month after then.

**Conclusions:**

The prevalence of GDM has sharply increased in the past decade. The growth rate of GDM slowed down after the implementation of the universal two-child policy in China, but the rate would maintain at a high plateau. The rise in the proportion of older pregnant women could increase the GDM rate. We recommend having children at a relatively optimal reproductive age when encouraging childbearing.

## Background

Gestational diabetes mellitus (GDM) is defined as impaired glucose tolerance (IGT) that first occurs or is first detected during pregnancy (1). It is an emerging epidemic (2, 3), and approximately one in six live births is associated with hyperglycemia exposure *in utero* according to the International Diabetes Federation (IDF) 2019 (4). The incidence of GDM ranges from 25% in South-East Asia to 17.5% in the Middle East and North Africa, 12.6% in Europe and 10.4% in North America and the Caribbean region (5).

Concurrently, the incidence of GDM has risen dramatically and has caused tremendous increases in medical expenditures in China in recent decades (6, 7). A recent systematic analysis of 79,064 pregnant women in 21 regions of mainland China reported a pooled GDM prevalence of 14.8% using the International Association of Diabetes and Pregnancy Study Group (IADPSG) diagnostic criteria (8). Of note, the incidence of GDM in China displayed substantial differences across cities and regions. For example, in the Tongzhou district of Beijing, the overall prevalence of GDM was 24.24%, and the trend increased from 21.63% to 25.49% during 2013-2018 (9). In the same period, a population-based study in Xiamen reported that the rate of GDM ranged from 15.5% to 19.9% (10). Another retrospective study conducted Guangzhou, China from 2011 through 2017 estimated a total GDM rate of approximately 22.94% (11). Similarly, 15.8% of pregnant women from four maternity hospitals in Chengdu, Western China, were diagnosed with GDM in 2015 (12). However, in some remote areas, such as Xinjiang, the estimated prevalence of GDM is only 5.12% (8), which might be due to the relatively lower rates of GDM screening during pregnancy.

Several reports proposed that the GDM incidence was increasing and accompanied by a high proportion of pregnant women with advanced maternal age after the implementation of the two-child policy (6, 13). In recent decades, China has gradually loosened fertility restrictions from the one-child policy to the partial two-child policy, universal two-child policy and even the three-child policy. The one-child policy was implemented in China for 36 years after its promulgation in 1979 (14), and the strategy was strictly enforced, particularly among urban residents; however, beginning in 1984, couples in rural areas were permitted to have a second baby if their first child was a girl, the so-called “1.5-child policy” (15). By 2007, all provinces in China started to pilot the two-child policy for couples in which both partners were only children, except for in Henan, where piloting began in 2011. Then, in 2013, couples in which at least one person was an only child were allowed to have two children (15). Later, in October 2015, the Chinese government encouraged all couples to have two children, which marked the official end of the one-child policy and the beginning of the new, universal two-child policy (15). The universal two-child policy targeted approximately 90 million reproductive-aged women who had delivered one baby; however, nearly 60% of the target women were over 35 years old, and 50% were over 40 (16). Thus, the projected increase in pregnant women with advanced maternal age and the consequent rise in pregnancy complications, such as GDM, after the implementation of the universal two-child policy have caused extensive concern.

Currently, data assessing trends in the prevalence of GDM in China before and after the implementation of the universal two-child policy are relatively scant. Our study aimed to identify the changes in GDM and related characteristics of pregnancy or delivery following the implementation of the universal two-child policy among 128,270 pregnant women who delivered at Ningbo Women & Children’s Hospital from January 2010 to December 2020.

## Materials and methods

### Study design and participants

This retrospective study was conducted to assess the temporal trend in GDM prevalence in women who delivered babies at Ningbo Women & Children’s Hospital from January 2010 through December 2020. A total of 140,676 pregnant women delivered their babies during this period. The exclusion criteria included pregestational diabetes mellitus (PGDM), duplicate records, and a missing diagnosis of GDM. A total of 128,270 eligible participants were eventually enrolled in the analysis. The data used in this study were extracted from electronic medical records.

### Data source

The data included sociodemographic characteristics, maternal disease status, obstetric history, pregnancy complications, mode of delivery, and maternal and neonatal delivery outcomes.

### Diagnosis of GDM

The fasting plasma glucose (FPG) test was carried out during the first trimester of gestation to leave out the pre-pregnancy diabetes, and the overt diabetes mellitus (DM) of FPG>7.0 mmol/L was excluded. GDM status was confirmed by two-step 50-g glucose challenge test (GCT) or one-step 75-g oral glucose tolerance test (OGTT) at 24-28 weeks of gestation. The 1-hour glucose value of 50-g GCT ≥11.2 mmol/L or 1-h glucose value ≥ 7.8 but subsequent 75-g OGTT had two or more items reached the standard criterions: FPG ≥ 5.6, 1h value ≥10.3, 2h value ≥8.6 or 3h value ≥ 6.7 was identified as GDM. The glucose value of one-step 75-g OGTT meeting or exceeding one of the following criteria: 0 h value ≥ 5.1 mmol/L, 1 h value ≥10.0 mmol/L, or 2 h value ≥ 8.5 mmol/L was diagnosed as GDM.

### Ethics approval

This study was approved by the Ningbo University Medical Science Research Ethics Committee.

### Statistical analysis

The major maternal and neonatal health characteristics are presented as the means ± standard deviations (SDs) or absolute frequencies (n) and relative frequencies (%) and were used to assess temporal trends from 2010 to 2020.

Segmented regression analyses of interrupted time series (ITS) were conducted to assess the effect of the two-child policy on the trend in GDM prevalence. The Durbin-Waston test was used to detect first-order autocorrelation (17), and the autocorrelated errors would be adjusted by the generalized least square estimator (GLSE) based on Prais-Winsten estimation. The ITS model was Y_t_ =β0 +β1*time _t_+ β2 *two-child policy _t_ +β3 *time after two-child policy _t_, where Y _t_ is the GDM rate per month t, and time t is a continuous variable representing the months since the start of the study. Two-child policy _t_ is a binary variable indicating the time before or after the implementation of the policy (coded as 0 for months between 2010 and 2016 and 1 for months thereafter). Time after two-child policy t is a continuous variable counting the number of months after the implementation of policy at time t. β0 is interpreted as the baseline level when T=0, and β1 indicates the preintervention slope. β2 and β3 present the change in the GDM rate after the intervention in the short- and long-term, respectively. The sum of β1 and β3 was used to evaluate the postintervention slope. The sensitivity analysis was conducted to estimate the robustness of ITS model using different month lags as the taking effect time after implementation of the universal two-chlid policy.

Univariate and multivariate logistic regression analysis were applied to estimate the potential risk factors of GDM, including reproductive age, gravidity, parity, the number of foetus and gestational hypertension (GH). Data analysis was carried out using SPSS software version 24.0 (SPSS Inc., Chicago, IL, USA) and R software (version 4.1.0). *P* values <0.05 for a two-tailed test were considered to be statistically significant.

## Results

A total of 128,270 eligible participants who delivered at Ningbo Women & Children’s Hospital between January 2010 and December 2020 were included, and the baseline characteristics and pregnancy outcomes are described in **Table 1**.

**Table:** Table 1 Characteristics of the participants according to delivery year.

Characteristics		2010	2011	2012	2013	2014	2015	2016	2017	2018	2019	2020
	N	9572	11,041	11,022	10,344	11,779	11,416	13,931	13,485	11,911	12,916	10,853
Age (year)		27±5	27±5	28±5	28±5	28±5	29±5	29±5	30±5	30±5	30±5	30±5
	<20	488 (5)	530 (5)	442 (4)	393 (4)	331 (3)	300 (3)	226 (2)	200 (2)	164 (1)	130 (1)	101 (1)
	20-	2667 (28)	2804 (25)	2560 (23)	2199 (21)	2048 (17)	1768 (16)	1756 (13)	1551 (12)	1362 (11)	1291 (10)	1043 (10)
	25-	3553 (37)	4450 (40)	4650 (42)	4371 (42)	5349 (45)	4971 (44)	6213 (45)	5409 (40)	4684 (39)	5176 (40.1)	4091 (38)
	30-	1844 (19)	2098 (19)	2371 (22)	2400 (23)	2914 (25)	3032 (27)	3888 (28)	3928 (29)	3591 (30)	4160 (32.2)	3724 (34)
	35-	854 (9)	905 (8)	798 (7)	789 (8)	950 (8)	1145 (10)	1581 (11)	1984 (15)	1751 (15)	1801 (13.9)	1596 (15)
	40-50	166 (2)	254 (2)	201 (2)	192 (2)	187 (2)	200 (2)	267 (2)	413 (3)	359 (3)	358 (2.8)	298 (3)
Gravidity (%)	One	3528 (37)	4442 (40)	4455 (40)	4261 (41)	4830 (41)	4177 (37)	5123 (37)	4346 (32)	4100 (34)	4692 (36.3)	3873 (36)
	Two	2656 (28)	2988 (27)	3006 (27)	2699 (26)	3129 (27)	3109 (27)	3873 (28)	3854 (29)	3269 (27)	3624 (28.1)	3012 (28)
	Three or more	3388 (35)	3611 (33)	3561 (32)	3384 (33)	3820 (32)	4130 (36)	4935 (35)	5285 (39)	4542 (38)	4600 (35.6)	3968 (37)
Parity (%)	Primipara	5839 (61)	7160 (65)	7198 (65)	6768 (65)	7613 (65)	6714 (59)	7910 (57)	6783 (50)	6244 (52)	6885 (53.3)	5836 (54)
	Multipara	3653 (38)	3756 (34)	3736 (34)	3524 (34)	4166 (35)	4702 (41)	6021 (43)	6702 (50)	5667 (48)	6031 (46.7)	5017 (46)
	NA	80 (1)	125 (1)	88 (1)	52 (1)	/	/	/	/	/	/	/
GDM (%)	Yes	337 (4)	429 (4)	878 (8)	1241 (12)	1772 (15)	1674 (15)	2285 (16)	2491 (19)	2088 (18)	2340 (18.1)	2271 (21)
	No	9235 (97)	10612 (96)	10144 (92)	9103 (88)	10007 (85)	9742 (85)	11646 (84)	10994 (82)	9823 (83)	10576 (81.9)	8582 (79)
GH (%)	Yes	496 (5)	515 (5)	532 (5)	458 (4)	527 (5)	455 (4)	434 (3)	430 (3)	421 (4)	578 (4.5)	574 (5)
	No	9076 (95)	10526 (95)	10490 (95)	9886 (96)	11252 (96)	10961 (96)	13497 (97)	13055 (97)	11490 (97)	12338 (95.5)	10279 (95)
Preeclampsia (%)	Yes	355 (4)	473 (4)	364 (3)	320 (3)	3 (0)	3 (0)	1 (0)	2 (0)	1 (0)	1 (0)	124 (1)
	No	9217 (96)	10568 (96)	10658 (97)	10024 (97)	11776 (100)	11413 (100)	13930 (100)	13483 (100)	11910 (100)	12915 (100)	10729 (99)
Polyhydramnios (%)	Yes	288 (3)	305 (3)	244 (2)	236 (2)	186 (2)	163 (1)	231 (2)	226 (2)	220 (2)	313 (2.4)	306 (3)
	No	9284 (97)	10736 (97)	10778 (98)	10108 (98)	11593 (98)	11253 (99)	13700 (98)	13259 (98)	11691 (98)	12603 (97.6)	10547 (97)
Oligohydramnios (%)	Yes	557 (6)	550 (5)	559 (5)	537 (5)	747 (6)	642 (6)	767 (6)	817 (6)	876 (7)	961 (7.4)	829 (8)
	No	9015 (94)	10491 (95)	10463 (95)	9807 (95)	11032 (94)	10774 (94)	13164 (95)	12668 (94)	11035 (93)	11955 (92.6)	10024 (92)
PROM (%)	Yes	1714 (18)	2159 (20)	2340 (21)	1982 (19)	2593 (22)	2381 (21)	3001 (22)	2816 (21)	2379 (20)	2624 (20.3)	2122 (20)
	No	7858 (82)	8882 (80)	8682 (79)	8362 (81)	9186 (78)	9035 (79)	10930 (79)	10669 (79)	9532 (80)	10292 (79.7)	8731 (80)
Placenta previa (%)	Yes	389 (4)	311 (3)	342 (3)	310 (3)	523 (4)	548 (5)	583 (4)	452 (3)	354 (3)	346 (2.7)	228 (2)
	No	9183 (96)	10730 (97)	10680 (97)	10034 (97)	11256 (96)	10868 (95)	13348 (96)	13033 (97)	11557 (97)	12570 (97.3)	10625 (98)
Number of foetus (%)	Single birth	9322 (97)	10663 (97)	10680 (97)	10021 (97)	11407 (97)	11016 (97)	13419 (96)	12968 (96)	11420 (96)	12398 (96)	10454 (96)
	Multiple births	250 (3)	378 (3)	342 (3)	323 (3)	372 (3)	400 (4)	512 (4)	517 (4)	491 (4)	518 (4)	399 (4)
Infant sex (%)	Male	5158 (54)	5802 (53)	5821 (53)	5490 (53)	6241 (53)	6062 (53)	7344 (53)	7196 (53)	6178 (52)	6842 (53)	5704 (53)
	Female	4333 (45)	5114 (46)	5110 (46)	4802 (46)	5537 (47)	5353 (47)	6586 (47)	6287 (47)	5729 (48)	6069 (47)	5144 (47)
	NA	81 (1)	125 (1)	91 (1)	52 (1)	1 (0)	1 (0)	1 (0)	2 (0)	4 (0)	5 (0)	5 (0)
Mode of delivery (%)	Vaginal birth	4303 (45)	4748 (43)	4869 (44)	4910 (48)	6041 (51)	5957 (52)	7215 (52)	7124 (53)	6272 (53)	6468 (50.1)	5248 (48)
	Cesarean	5007 (52)	5959 (54)	5772 (52)	5138 (50)	5470 (46)	5188 (45)	6296 (45)	6125 (45)	5486 (46)	6133 (47.5)	5110 (47)
	NA	262 (3)	334 (3)	381 (4)	296 (3)	268 (2)	271 (2)	420 (3)	236 (2)	153 (1)	315 (2.4)	495 (5)
Full-term birth (%)	Yes	8431 (88)	9500 (86)	9364 (85)	8970 (87)	10180 (86)	9975 (87)	12176 (87)	11846 (88)	10518 (88)	11352 (87.9)	9532 (88)
	No	1141 (12)	1541 (14)	1658 (15)	1374 (13)	1599 (14)	1441 (13)	1755 (13)	1639 (12)	1393 (12)	1564 (12.1)	1321 (12)
Pregnancy outcomes (%)	Livebirth	9530 (100)	10995 (100)	10985 (100)	10267 (99)	11673 (99)	11326 (99)	13857 (100)	13409 (99)	11853 (100)	12837 (99.4)	10815 (100)
	Malformation	8 (0)	14 (0)	2 (0)	19 (0)	8 (0)	10 (0)	4 (0)	2 (0)	7 (0)	18 (0.1)	9 (0)
	Stillbirth	34 (0)	32 (0)	35 (0)	46 (0)	90 (1)	73 (1)	70 (1)	70 (1)	50 (0)	57 (0.4)	29 (0)
	NA	/	/	/	12 (0)	8 (0)	7 (0)	/	4 (0)	1 (0)	4 (0)	/
Macrosomia (%)	Yes	250 (3)	208 (2)	206 (2)	193 (2)	660 (6)	712 (6)	869 (6)	830 (6)	646 (5)	700 (5.4)	567 (5)
	No	9322 (97)	10833 (98)	10816 (98)	10151 (98)	11119 (94)	10704 (94)	13062 (94)	12655 (94)	11265 (95)	12216 (94.6)	10286 (95)

NA, not available; GDM, Gestational diabetes mellitus; GH, Gestational hypertension; PROM, premature rupture of membranes.

The mean age at delivery increased from 27 years (2010) to 30 years (2020). In the age stratification, pregnant women aged 25-29 years accounted for the major proportion during the examined years. The proportion of women aged 20-24 years decreased from 28% to 10%, while women aged 30-34 years and ≥35 years (advanced maternal age) increased from 19% to 34% and 9% to 15%, respectively. In addition, the proportions of primiparas and multiparas have gradually become comparable since 2017 (**Table 1**).

Univariate logistic regression analysis found associations of advanced maternal age [30-34 years, *OR* 1.64 (1.58-1.70); 35-39 years, *OR* 2.54 (2.42-2.66); 40-50 years, *OR* 3.26 (2.99-3.54)], multigravidity [two times, *OR* 1.18 (1.13-1.23); ≥3 times, *OR* 1.48 (1.43-1.54)], multipara [*OR* 1.27 (1.23-1.31)], multiple gestation [*OR* 1.46 (1.35-1.57)] and GH [*OR* 1.69 (1.58-1.81)] with GDM (**Table 2**). These variables were all included in the multivariate logistic regression model, and the age, multigravidity, multiple gestation and GH remained the significant risk factors for GDM, but the multipara became negative correlation wuth GDM (**Table 2**). The proportion of advanced maternal age (≥30 years) was highly distributed in women with two or more pregnancy (51%) or multipara (61%) compared with first pregnancy (20%) or primipara (24%) (**Table 3**). Subgroup analysis found that age (≥30 years) remained the strongest risk factor for GDM even after stratification with gravidity or parity (**Table 3**).

**Table:** Table 2 Univariate and multivariate logistic regression analysis of factors associated with GDM.

Characteristics	Group	GDM	non-GDM	*OR*(95%*CI*)	*P*
Univariate logistic regression analysis					
Age	25-29	6105 (34)	46812 (42)	Reference	
	<20	98 (1)	3207 (3)	0.23 (0.19–0.29)	<0.001
	20-24	1242 (7)	19807 (18)	0.48 (0.45–0.51)	<0.001
	30-34	5976 (34)	27974 (25)	1.64 (1.58–1.70)	<0.001
	35-39	3522 (20)	10632 (10)	2.54 (2.42–2.66)	<0.001
	40-50	863 (5)	2032 (2)	3.26 (2.99–3.54)	<0.001
Gravidity	One	5605 (32)	42222 (38)	Reference	
	Two	4754 (27)	30465 (28)	1.18 (1.13–1.23)	<0.001
	Three or more	7447 (42)	37777 (34)	1.48 (1.43–1.54)	<0.001
Parity	Primipara	9530 (54)	65420 (59)	Reference	
	Multipara	8267 (46)	44708 (41)	1.27 (1.23–1.31)	<0.001
Multiple gestation	No	16960 (95)	106808 (97)	Reference	
	Yes	846 (5)	3656 (3)	1.46 (1.35–1.57)	<0.001
GH	No	16670 (94)	106180 (96)	Reference	
	Yes	1136 (6)	4284 (4)	1.69 (1.58–1.81)	<0.001
**Multivariate logistic regression**					
Age				1.12 (1.11–1.12)	<0.001
Gravidity				1.07 (1.02–1.12)	0.004
Multipara				0.80 (0.77–0.84)	<0.001
Multiple gestation				1.35 (1.25–1.46)	<0.001
GH				1.46 (1.36–1.56)	<0.001

**Table:** Table 3 Stratification analysis by age and gravidity/parity for GDM.

Subgroups	Age>30y stratification (%)	GDM	non-GDM	*OR*(95%*CI*)	*P*
Gravidity & Age					
<30 & one pregnancy	38163 (80)	3660 (21)	34503 (31)	Reference	
≥30 & one pregnancy	9664 (20)	1945 (11)	7719 (7)	2.38 (2.24-2.52)	<0.001
<30 & two or more pregnancy	39108 (49)	3785 (21)	35323 (32)	1.01 (0.96-1.06)	0.68
≥30 & two or more pregnancy	41335 (51)	8416 (47)	32919 (30)	2.41 (2.31-2.51)	<0.001
Parity & Age					
<30 & Primipara	56685 (76)	5623 (32)	51062 (46)	Reference	
≥30 & Primipara	18265 (24)	3907 (22)	14358 (13)	2.47 (2.36–2.58)	<0.001
<30 & Multipara	20444 (39)	1817 (10)	18627 (17)	0.89 (0.84–0.94)	<0.001
>30 & Multipara	32531 (61)	6450 (36)	26081 (24)	2.25 (2.16–2.33)	<0.001

GDM; Gestational diabetes mellitus.

**Figure 1:**
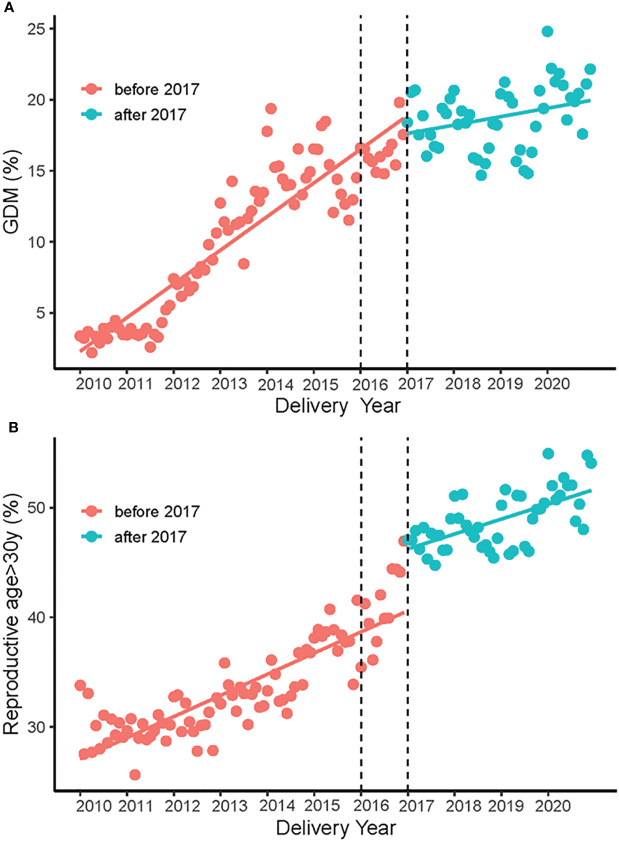
The increased trend of GDM rate and the proportion of women with advanced maternal age (>30 years) from 2010 to 2020 **(A)**. The growth trend of GDM before and after the implementation of the universal two-child policy **(B)**. The growth trend of the proportion of women with advanced maternal age (>30 years) before and after the implementation of the universal two-child policy.

The prevalence of GDM increased considerably from 4% in 2010 to 21% in 2020 (**Table 1**). **Figure 1A** displays a sharply increased GDM rate from 2010 to 2017, a relatively slow growth trend maintained after 2017. ITS analysis was applied to assess the change of the increased trend of GDM after the universal two-child policy, and the results illustrated that the prevalence of GDM increased by 0.190% (β1) per month from 2010 to 2016 (*P*<0.05), but by 0.044% (β1+β3) per month after the implementation of the universal two-child policy since 2017; the change of the trend was statistically significant (β3=-0.146, *P*=0.004) (**Supplementary Table 1**). Moreover, sensitive analysis were performed by setting different time lags after the implementation of policy, and ITS analysis at different month lags showed the same effect of the universal two-child policy on the change of GDM (**Supplementary Table 2**). We further assessed the effect of the universal two-child policy on the change of reproductive age. The results showed that the proportion of women with advanced maternal age (>30 years) increased by 0.161% per month before the implementation of the universal two-child policy, increased by 5.25% during the policy took effect month (set in January 2017), and then gradually increased by 0.124% (β1+β3) per month after then(**Figure 1B** and **Supplementary Table 1**).

**Figure 2:**
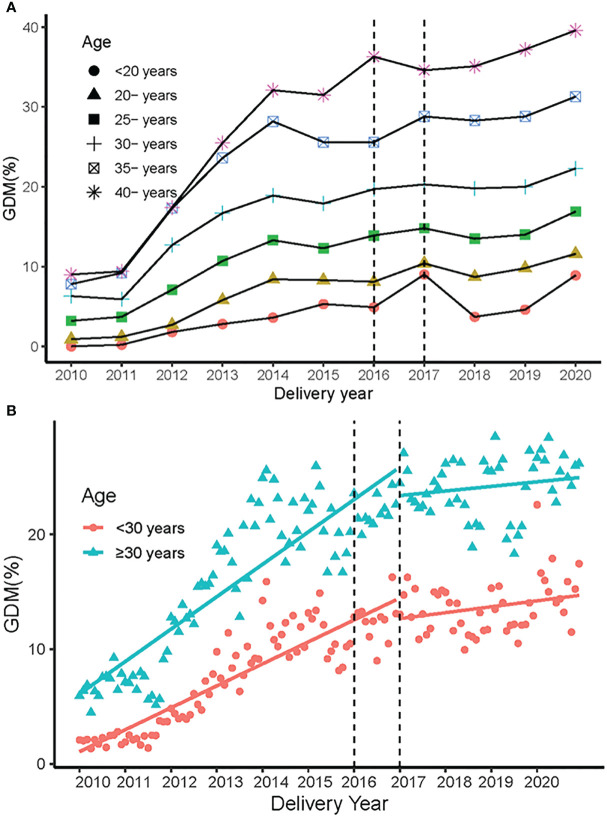
The prevalence of GDM in different age groups in 2010-2020. **(A)** The prevalence of GDM in different age groups from 2010 to 2020. **(B)** The change in growth trend of GDM before and after the universal two-child policy in different age groups.

**Figure 3:**
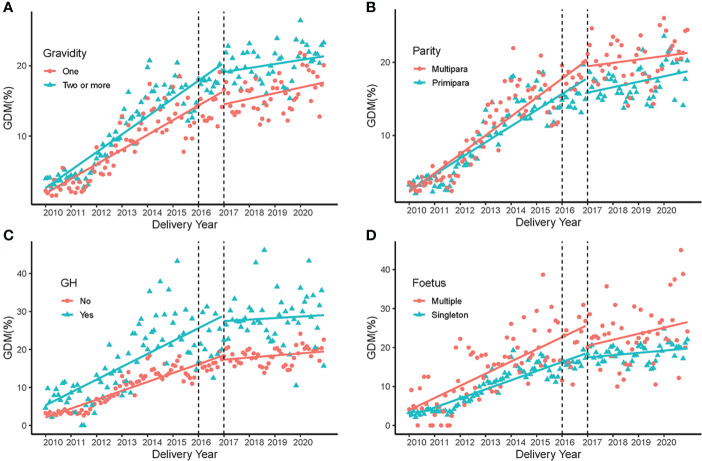
GDM rate in 2010-2020 in different subgroups. **(A)** Trends of GDM rate in different gravidity groups before and after the the universal two-child policy. **(B)** Trends of GDM rate in the primipara and multipara groups before and after the universal two-child policy. **(C)** Trends of GDM rate in women with and without gestational hypertension before and after the universal two-child policy. **(D)** Trends of GDM rate in the singleton and multiple fetus groups before and after the universal two-child policy.

**Figures 2A, B** presented the increased trend of GDM in different age groups, the women aged older than 30 years had a higher prevalence of GDM. Similarly, **Figures 3A–D** showed a higher GDM rate in women with multigravidity, multiparity, multiply gestations or GH. The rate of elevation of GDM slowed down in all groups after the two-chlid policy implemented in 2017 **(** **Supplementary Table 1** **)**.

## Discussion

The current study showed that the prevalence of GDM increased rapidly from 4% in 2010 to 21% in 2020 based on data from 128,270 pregnant women who delivered at Ningbo Women & Children’s Hospital. The prevalence of GDM sharply increased, particularly from 2010 to 2016, and then maintained a slow growth trend since 2017. ITS analysis also demonstrated that the rate of elevation of GDM slowed down after the implementation of the universal two-child policy. Furthermore, our study found that the risk of GDM was markedly increased with advanced maternal age. It was noted that the maternal age at childbirth increased by an average of 3 years from 2010 to 2020. Moreover, the proportion of pregnant women older than 30 years increased, ranging from 29.9% (2010) to 51.7% (2020), and the growth trend in reproductive age (>30 years) still increased by 0.124% per month after the implementation of the universal two-child policy since 2017. These results indicated that the rising proportion of older pregnant women resulting from the implementation of the universal two-child policy might be an independent risk factor for GDM.

Our findings were generally consistent with several large population-based studies that demonstrated a strong relationship between advanced maternal age and GDM (18-20). Two large multicenter cohort investigations of singleton pregnancies reported that the GDM rate increased with maternal age (18, 19), and the rate reached a high plateau at approximately 40 years of age (18). Another prospective study based on national registry data also proposed that women aged 40-44 years and those aged 45 years or older had nearly three- and fourfold increased risks of developing GDM, respectively (20). Moreover, Chantal Mathieu et al. indicated that screening GDM by a maternal age ≥30 years and/or a BMI ≥25 could identify 81% of cases based on the 2013 WHO criteria (21). The underlying mechanism could be explained by the association of aging with reduced insulin sensitivity, abnormal glucose tolerance, and impaired pancreatic B-cell function (18). However, the data from the Diabetes Surveillance System of the Ningbo Center for Disease Control and Prevention (CDC) showed that the age at GDM diagnosis was markedly increased after 2016 in Ningbo city, but the BMI was relatively steady. It seemed that delayed childbearing age was the most important risk factor for GDM after the implementation of the universal two-child policy.

In the current study, the rise in the proportion of older pregnant women might be maintained by the change in fertility policy. In particular, women aged 30-34 years accounted for the overwhelming majority among the increased proportion of older women (>30 years) and presented an increasing trend after the implementation of the two-child policy. However, the proportion of women aged 40 years or older remained lower than 3%. Accordingly, we speculate that eligible couples who desired to have children and were younger than 35 years old were more likely to apply for a second child after the implementation of the universal two-child policy, but the majority of women older than 40 years would express a lower desire for a second child given the potential for birth defects. In addition, several social factors, such as delayed marriage, the rising ratio of divorce and remarriage rates, increased desires for higher education and career achievement, the development of assisted reproductive technology and growing financial burdens, could contribute to postponing childbearing in China. Therefore, we advocate for having children at a relatively optimal reproductive age when encouraging childbearing.

Although advanced maternal age was a strong independent risk factor for GDM, other physiological and socioeconomic factors should also be fully considered. Among our findings, multigravidity, multiparity, multiple gestation and gestational hypertension also contributed to GDM incidence, although these factors exerted less effects than age, and the trend in GDM exhibited slower growth in the above exposure groups after the implementation of the two-child policy in 2017. Previous research proposed an association between abortion and GDM (22). Our study found that the proportion of women with three or more pregnancies remained at approximately 35% in the past decade. It is not difficult to guess that most of these women had undergone induced abortions. The two-child policy could substantially reduce the number of abortions due to sex selection and unapproved second children, which would greatly improve maternal and infant adverse outcomes (15).

The trend of rapid growth in the prevalence of GDM during the past decade was also linked with elevated GDM screening, enhanced awareness of prenatal health care, improved medical service systems and modifiable lifestyle factors (23, 24). The diagnostic criteria of GDM using the IADPSG (2010) have been gradually recommended in China since 2011. The 2011 edition of the GDM health industry standards by the Ministry of Health of China, the 2013 edition of the Chinese Guidelines for the Diagnosis and Treatment of Diabetes Mellitus, and the Obstetrics and Gynecology Subcommittee of the Chinese Medical Association 2014 all adopted the IADPSG standard. These criteria recommend a “one-step” method (75 g OGTT) of testing at 24-28 weeks of gestation and have helped to identify more cases of GDM in pregnant women (25, 26). In our study, the one-step 75-g OGTT method almost completely replaced the two-step 50-g GCT method in Ningbo, Zhejiang province, China since 2012. Herein, the rapid rise in GDM in our study should be partially due to improved screening and diagnostic technology. Moreover, delayed childbearing age and the accompanying increase in assisted reproduction technology use can also exacerbate the incidence of GDM (27, 28). Other factors, such as genetic variations, obesity, inappropriate lifestyles during pregnancy and environmental exposures, are also associated with GDM (6, 29, 30). However, since the implementation of the policy to encourage childbirth, women of childbearing age have paid more attention to reproductive health and prenatal care, and improved health systems would provide more guidance and intervention before and during pregnancy, all of which might effectively reduce the occurrence of GDM. Taken together, the universal two-child policy might not aggravate the prevalence of GDM, but the GDM rate could remain at a relatively stable high level in the future.

In fact, most couples of childbearing age are cautious about the second-child and third-child policies. In November 2013, China launched the partial two-child policy allowing couples to have a second child when at least one member of the couple was an only child. However, by May 2015, only 13.2% (1.45/11 million) of eligible couples wanted to have a second child (15). Shortly afterward, the universal two-child policy was announced (October 2015). The number of live births reached 17.86 million and 17.23 million in 2016 and 2017 in China (31, 32), respectively, increasing by 7.9% and 4.1% compared with 2015 (**Supplementary Figure 1**); in particular, approximately 5.4 million births of multiparous women during the first eighteen months were due to the new policy (16). Surprisingly, China’s fertility level did not increase continuously, as expected, and the numbers of live births in 2018 and 2019 were only 15.23 million and 14.65 million, respectively, which were even lower than the birth rates before the implementation of the universal two-child policy (**Supplementary Figure 1**) (33, 34). Consistently, our data also showed an apparent fertility peak in 2016 and 2017 and then a decline from the peak. Furthermore, the China Family Panel Studies (CFPS) 2018 reported that less than 10% of men and women aged 18-49 years present the desire to have a third child, of which 4.90% of those aged 18-24 years desire to have a third child, 6.92% of those aged 25-29 years desire to have a third child, and 16% of those aged 45-49 years desire to have a third child. Consequently, the lower fertility desire and higher proportion of pregnant women with advanced maternal age are still inevitable trends even after the three-child policy. Hence, the long-term impact of the fertility policy on fertility desire and childbearing age needs further observation in the future.

Our study focused on the prevalence of GDM and associated risk factors across the implementation of the universal two-child policy from 2010 to 2020. This provided relatively reliable evidence of maintaining an upward trend in GDM with advanced reproductive age after the implementation of the universal two-child policy. Nevertheless, our data were limited to a single center: the Ningbo Women & Children’s Hospital, Zhejiang Province. Hence, the results might vary in other districts in China due to different living habits, environmental exposures, and socioeconomic development.

## Conclusions

In conclusion, the present study found that the prevalence of GDM has sharply increased in the past decade. Although, the growth rate of GDM slowed down after implementation of the universal two-child policy, the rate would maintain at a high plateau in the future. Notably, advanced maternal age was an independent risk factor for GDM, and the remarkable rise in the proportion of older pregnant women with the change in fertility policy seemed to be associated with the prevalence of GDM, as expected. In the future, large multicenter cohort studies are warranted to determine the long-term impact of the universal two-child policy on GDM.

## Data availability statement

The original contributions presented in the study are included in the article/**Supplementary Material**. Further inquiries can be directed to the corresponding authors.

## Ethics statement

The studies involving human participants were reviewed and approved by Ningbo University Medical Science Research Ethics Committee. Written informed consent for participation was not required for this study in accordance with the national legislation and the institutional requirements.

## Author contributions

LJ conceived the study with HZ and JX. JC, ZZ, YC, QZ, and LZ contributed to the acquisition of data, and HZ, ZZ, YC, LJ, and JX contributed to the analysis and interpretation of the data. HZ, LJ, and JX drafted the initial article, and all co-authors contributed to revising it for intellectual content. All co-authors have given final approval of the submitted version. The corresponding author attests that all listed authors meet authorship criteria and that no others meeting the criteria have been omitted. LJ is the guarantor. All authors contributed to the article and approved the submitted version.

## Funding

This research was supported by the Zhejiang Public Welfare Technology Application Research Program (LGF20H260009, LGF20H040005), Zhejiang Medical and Health Science and Technology Program (2019KY648), Ningbo Nonprofit Science and Technology Project (2019C50097, 2021S132), and Ningbo Medical and Health Brand Discipline (PPXK2018-06)

## Conflict of interest

The authors declare that the research was conducted in the absence of any commercial or financial relationships that could be construed as a potential conflict of interest.

## Publisher’s note

All claims expressed in this article are solely those of the authors and do not necessarily represent those of their affiliated organizations, or those of the publisher, the editors and the reviewers. Any product that may be evaluated in this article, or claim that may be made by its manufacturer, is not guaranteed or endorsed by the publisher.

## Supplementary material

Supplementary Figure 1 Total births in mainland China and total delivery volume in Ningbo Women & Children’s Hospital from 2010 to 2020 The bar graph corresponds to the left ordinate value, and the line graph corresponds to the right ordinate value. The data for the total number of births and the total birth rate in mainland China from 2010-2020 were extracted from the National Economic and Social Development Statistical Bulletin of the People’s Republic of China.
