# Supplementary figures and images for: The prevalence of gestational diabetes mellitus before and after the implementation of the universal two-child policy in China
Source: Front Endocrinol (Lausanne). 2022 Aug 18;13:960877. doi: 10.3389/fendo.2022.960877 (PMC9433653; doi:10.3389/fendo.2022.960877)

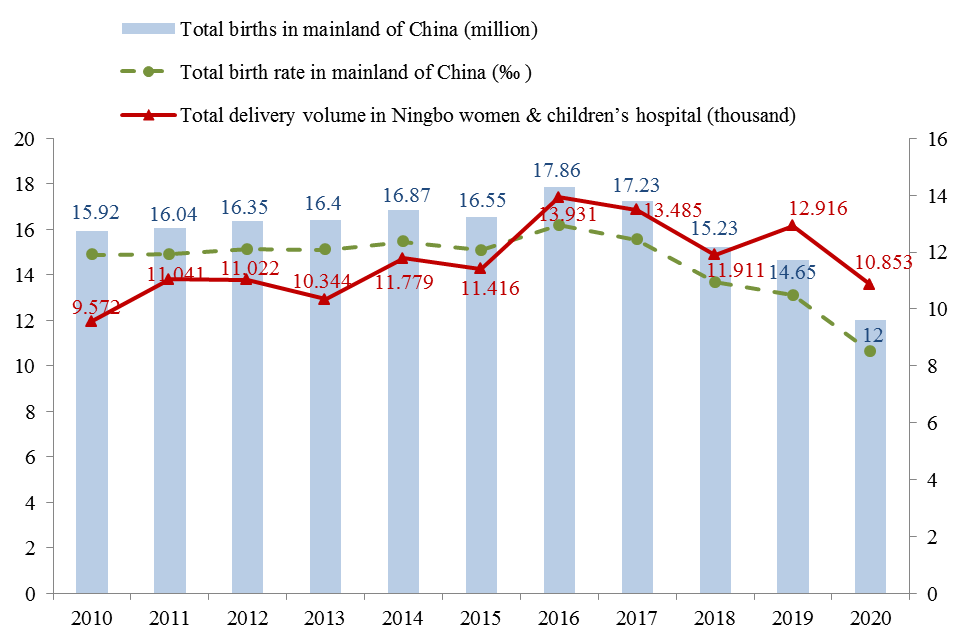

Supplement: Supplementary Figure 1 — Total births in mainland China and total delivery volume in Ningbo Women & Children’s Hospital from 2010 to 2020 The bar graph corresponds to the left ordinate value, and the line graph corresponds to the right ordinate value. The data for the total number of births and the total birth rate in mainland China from 2010-2020 were extracted from the National Economic and Social Development Statistical Bulletin of the People’s Republic of China. [file Image_1.tif]
