# Supplementary material for: The prevalence of gestational diabetes mellitus before and after the implementation of the universal two-child policy in China
Source: Front Endocrinol (Lausanne). 2022 Aug 18;13:960877. doi: 10.3389/fendo.2022.960877 (PMC9433653; doi:10.3389/fendo.2022.960877)
Supplement: Supplementary file 2 [file Table_1.docx]

Supplementary Table 1 Interrupted time series model to determine impacts of the universal two-child policy on the trend of GDM

|  | Coefficients | Estimate | Std. Error | t value | Pr(>\|t\|) | D-W Statistic (original) | D-W Statistic (transformed) ^#^ |
| --- | --- | --- | --- | --- | --- | --- | --- |
| **Total GDM** |  |  |  |  |  |  |  |
| (Intercept) | β0 | 2.373 | 1.010 | 2.35 | 0.02030 * | 0.66 | 2.22 |
| x1 | β1 | 0.190 | 0.020 | 9.52 | < 2e-16 *** |  |  |
| x2 | β2 | -0.482 | 1.276 | -0.38 | 0.71 |  |  |
| x3 | β3 | -0.146 | 0.050 | -2.95 | 0.00383 ** |  |  |
| **Reproductive age** |  |  |  |  |  |  |  |
| (Intercept) | β0 | 26.983 | 0.686 | 39.35 | < 2e-16 *** | 1.32 | 2.07 |
| x1 | β1 | 0.161 | 0.014 | 11.56 | < 2e-16 *** |  |  |
| x2 | β2 | 5.250 | 1.063 | 4.94 | 2.39e-06 *** |  |  |
| x3 | β3 | -0.037 | 0.034 | -1.07 | 0.29 |  |  |
| **GDM in <30 years group** |  |  |  |  |  |  |  |
| (Intercept) | β0 | 0.984 | 0.710 | 1.39 | 0.17 | 1.13 | 2.21 |
| x1 | β1 | 0.157 | 0.014 | 10.93 | < 2e-16 *** |  |  |
| x2 | β2 | -1.622 | 1.070 | -1.52 | 0.13 |  |  |
| x3 | β3 | -0.113 | 0.035 | -3.20 | 0.00175 ** |  |  |
| **GDM in >30 years group** |  |  |  |  |  |  |  |
| (Intercept) | β0 | 6.169 | 1.360 | 4.54 | 1.29e-05 *** | 0.70 | 2.33 |
| x1 | β1 | 0.224 | 0.027 | 8.32 | 1.08e-13 *** |  |  |
| x2 | β2 | -0.869 | 1.757 | -0.50 | 0.62 |  |  |
| x3 | β3 | -0.216 | 0.067 | -3.24 | 0.00154 ** |  |  |
|  |  |  |  |  |  |  |  |
| **GDM in gravidity one** |  |  |  |  |  |  |  |
| (Intercept) | β0 | 1.78792 | 0.78687 | 2.272 | 0.02473 * | 1.20 | 2.13 |
| x1 | β1 | 0.16983 | 0.01596 | 10.642 | < 2e-16 *** |  |  |
| x2 | β2 | -1.31747 | 1.1896 | -1.107 | 0.27014 |  |  |
| x3 | β3 | -0.10863 | 0.03925 | -2.768 | 0.00647 ** |  |  |
| **GDM in gravidity≥2 times** |  |  |  |  |  |  |  |
| (Intercept) | β0 | 2.60039 | 1.02265 | 2.543 | 0.0122 * | 0.78 | 2.28 |
| x1 | β1 | 0.20728 | 0.02039 | 10.166 | <2e-16 *** |  |  |
| x2 | β2 | -0.84244 | 1.38479 | -0.608 | 0.544 |  |  |
| x3 | β3 | -0.15804 | 0.05034 | -3.14 | 0.0021 ** |  |  |
| **GDM in primipara** |  |  |  |  |  |  |  |
| (Intercept) | β0 | 2.465 | 0.873 | 2.82 | 0.00551 ** | 0.94 | 2.17 |
| x1 | β1 | 0.178 | 0.018 | 10.14 | < 2e-16 *** |  |  |
| x2 | β2 | -1.398 | 1.256 | -1.11 | 0.27 |  |  |
| x3 | β3 | -0.124 | 0.043 | -2.86 | 0.00494 ** |  |  |
| **GDM in multipara** |  |  |  |  |  |  |  |
| (Intercept) | β0 | 2.181 | 0.974 | 2.24 | 0.02687 * | 1.02 | 2.29 |
| x1 | β1 | 0.212 | 0.020 | 10.79 | < 2e-16 *** |  |  |
| x2 | β2 | -0.637 | 1.434 | -0.44 | 0.66 |  |  |
| x3 | β3 | -0.172 | 0.048 | -3.55 | 0.00053 *** |  |  |
|  |  |  |  |  |  |  |  |
| **GDM in one Foetus** |  |  |  |  |  |  |  |
| (Intercept) | β0 | 2.336 | 0.996 | 2.35 | 0.02057 * | 0.69 | 2.24 |
| x1 | β1 | 0.187 | 0.020 | 9.50 | < 2e-16 *** |  |  |
| x2 | β2 | -0.384 | 1.288 | -0.30 | 0.77 |  |  |
| x3 | β3 | -0.145 | 0.049 | -2.96 | 0.00371 ** |  |  |
| **GDM in two or more foetuses** |  |  |  |  |  |  |  |
| (Intercept) | β0 | 3.674 | 1.547 | 2.38 | 0.0190 * | 1.79 | 1.98 |
| x1 | β1 | 0.260 | 0.032 | 8.24 | 1.66e-13 *** |  |  |
| x2 | β2 | -5.357 | 2.453 | -2.18 | 0.0308 * |  |  |
| x3 | β3 | -0.132 | 0.078 | -1.69 | 0.0932 . |  |  |
| **GDM in group without GH** |  |  |  |  |  |  |  |
| (Intercept) | β0 | 2.167 | 0.954 | 2.27 | 0.02480 * | 0.71 | 2.15 |
| x1 | β1 | 0.188 | 0.019 | 9.94 | < 2e-16 *** |  |  |
| x2 | β2 | -0.548 | 1.246 | -0.44 | 0.66 |  |  |
| x3 | β3 | -0.143 | 0.047 | -3.05 | 0.00278 ** |  |  |
| **GDM in group without GH** |  |  |  |  |  |  |  |
| (Intercept) | β0 | 5.052 | 1.734 | 2.91 | 0.00421 ** | 1.61 | 2.03 |
| x1 | β1 | 0.281 | 0.035 | 7.95 | 8.31e-13 *** |  |  |
| x2 | β2 | -1.285 | 2.732 | -0.47 | 0.64 |  |  |
| x3 | β3 | -0.258 | 0.087 | -2.96 | 0.00368 ** |  |  |
| <0.00001 ‘***’ <0.001 ‘**’ <0.01 ‘*’ | |  |  |  |  |  |  |

Segmented regression analyses of interrupted time series (ITS) were conducted to assess the effect of the two-child policy on the trend in GDM prevalence. The Durbin-Waston test was used to detect first-order autocorrelation (23), and the autocorrelated errors would be adjusted by the generalized least square estimator (GLSE) based on Prais-Winsten estimation would be used to conduct the model.

β0 is interpreted as the baseline level when T=0, and β1 indicates the pre-intervention slope. β2 and β3 present the change in the GDM rate after the intervention in the short- and long-term, respectively. The sum of β1 and β3 was used to evaluate the post-intervention slope.

**Supplementary Table 2** Interrupted time series model to determine the impact of the universal two-child policy on the trend of total GDM using different month lags

|  | Coefficients | Estimate | Std. Error | t value | Pr(>\|t\|) | D-W Statistic (original) | D-W Statistic (transformed) |
| --- | --- | --- | --- | --- | --- | --- | --- |
| Start at 9 months after the policy |  |  |  |  |  |  |  |
| (Intercept) | β0 | 2.00173 | 1.02297 | 1.957 | 0.0525 . | 0.66 | 2.185 |
| x1 | β1 | 0.2037 | 0.0217 | 9.389 | 2.81e-16 *** |  |  |
| x2 | β2 | -1.18104 | 1.26521 | -0.933 | 0.3523 |  |  |
| x3 | β3 | -0.13129 | 0.04414 | -2.974 | 0.0035 ** |  |  |
| Start at 10 months after the policy |  |  |  |  |  |  |  |
| (Intercept) | β0 | 2.30173 | 1.03183 | 2.231 | 0.02743 * | 0.668 | 2.203 |
| x1 | β1 | 0.19192 | 0.02155 | 8.904 | 4.27e-15 *** |  |  |
| x2 | β2 | -0.04745 | 1.26416 | -0.038 | 0.97012 |  |  |
| x3 | β3 | -0.14404 | 0.04562 | -3.158 | 0.00198 ** |  |  |
| Start at 11 months after the policy |  |  |  |  |  |  |  |
| (Intercept) | β0 | 2.23764 | 1.01811 | 2.198 | 0.02975 * | 0.6618 | 2.193 |
| x1 | β1 | 0.19441 | 0.02103 | 9.245 | 6.33e-16 *** |  |  |
| x2 | β2 | -0.49359 | 1.26009 | -0.392 | 0.69592 |  |  |
| x3 | β3 | -0.14191 | 0.04587 | -3.094 | 0.00242 |  |  |
| Start at 12 months after the policy |  |  |  |  |  |  |  |
| (Intercept) | β0 | 2.12272 | 1.02156 | 2.078 | 0.03970 * | 0.6499 | 2.177 |
| x1 | β1 | 0.19892 | 0.02084 | 9.546 | < 2e-16 *** |  |  |
| x2 | β2 | -1.41929 | 1.26566 | -1.121 | 0.26421 |  |  |
| x3 | β3 | -0.131 | 0.04693 | -2.791 | 0.00605 ** |  |  |
| Start at 13 months after the policy |  |  |  |  |  |  |  |
| (Intercept) | β0 | 2.71349 | 1.127 | 2.408 | 0.01747 * | 0.657 | 2.198 |
| x1 | β1 | 0.17671 | 0.02256 | 7.834 | 1.53e-12 *** |  |  |
| x2 | β2 | 1.72157 | 1.32853 | 1.296 | 0.19734 |  |  |
| x3 | β3 | -0.16693 | 0.0527 | -3.168 | 0.00192 ** |  |  |
| Start at 14 months after the policy |  |  |  |  |  |  |  |
| (Intercept) | β0 | 2.08775 | 1.01362 | 2.06 | 0.04144 * |  |  |
| x1 | β1 | 0.20002 | 0.0202 | 9.902 | < 2e-16 *** |  |  |
| x2 | β2 | -1.82581 | 1.26842 | -1.439 | 0.15245 |  |  |
| x3 | β3 | -0.12994 | 0.04856 | -2.676 | 0.00843 ** |  |  |

Segmented regression analyses of interrupted time series (ITS) were conducted to assess the effect of the two-child policy on the trend in GDM prevalence. The Durbin-Waston test was used to detect first-order autocorrelation (23), and the autocorrelated errors would be adjusted by the generalized least square estimator (GLSE) based on Prais-Winsten estimation would be used to conduct the model.

β0 is interpreted as the baseline level when T=0, and β1 indicates the pre-intervention slope. β2 and β3 present the change in the GDM rate after the intervention in the short- and long-term, respectively. The sum of β1 and β3 was used to evaluate the post-intervention slope.
